# Advance of soy commodity in the southern Amazonia with deforestation via PRODES and ImazonGeo: a moratorium-based approach

**DOI:** 10.1038/s41598-021-01350-y

**Published:** 2021-11-08

**Authors:** Thais Lourençoni, Carlos Antonio da Silva Junior, Mendelson Lima, Paulo Eduardo Teodoro, Tatiane Deoti Pelissari, Regimar Garcia dos Santos, Larissa Pereira Ribeiro Teodoro, Iago Manuelson Luz, Fernando Saragosa Rossi

**Affiliations:** 1 State University of Mato Grosso (UNEMAT), Alta Floresta, MT Brazil; 2 Department of Geography, State University of Mato Grosso (UNEMAT), Sinop, MT Brazil; 3 Department of Crop Science, Department of Agronomy, Federal University of Mato Grosso Do Sul (UFMS), Chapadão Do Sul, MS Brazil; 4 State University of São Paulo (UNESP), Ilha Solteira, SP Brazil; 5 State University of São Paulo (UNESP), Jaboticabal, SP Brazil

**Keywords:** Environmental sciences, Natural hazards

## Abstract

The guidance on decision-making regarding deforestation in Amazonia has been efficient as a result of monitoring programs using remote sensing techniques. Thus, the objective of this study was to identify the expansion of soybean farming in disagreement with the Soy Moratorium (SoyM) in the Amazonia biome of Mato Grosso from 2008 to 2019. Deforestation data provided by two Amazonia monitoring programs were used: PRODES (Program for Calculating Deforestation in Amazonia) and ImazonGeo (Geoinformation Program on Amazonia). For the identification of soybean areas, the Perpendicular Crop Enhancement Index (PCEI) spectral model was calculated using a cloud platform. To verify areas (polygons) of largest converted forest-soybean occurrences, the Kernel Density (KD) estimator was applied. Mann–Kendall and Pettitt tests were used to identify trends over the time series. Our findings reveal that 1,387,288 ha were deforested from August 2008 to October 2019 according to PRODES data, of which 108,411 ha (7.81%) were converted into soybean. The ImazonGeo data showed 729,204 hectares deforested and 46,182 hectares (6.33%) converted into soybean areas. Based on the deforestation polygons of the two databases, the KD estimator indicated that the municipalities of Feliz Natal, Tabaporã, Nova Ubiratã, and União do Sul presented higher occurrences of soybean fields in disagreement with the SoyM. The results indicate that the PRODES system presents higher data variability and means statistically superior to ImazonGeo.

## Introduction

Amazonia extends over nine countries in South America, with about 60% of its area within Brazilian territory. It is recognized worldwide for its beauty and biodiversity, acting in the regulation of the local, regional, and global climate^1–6^.

Despite its exuberance and the ecosystem services it provides, the forest has been constantly affected by deforestation, resulting in biodiversity loss and climate change^2–4,7^. Ecological modeling assessments indicate that if deforestation exceeds 20–40%, the Amazonia climate and vegetation will reach its tipping point^8–10^. Today, the forest has already lost 20% of its original vegetation cover, which has been replaced by pastures and soybean crops^11–13^.

One of the ways to contain deforestation in the region were the agreements signed, such as the Soy Moratorium (SoyM) in the soybean sector, which foresees the non-commercialization of soybeans originating from deforested areas as from August 2008, and the Beef Conduct Adjustment Terms (TAC) in the livestock sector^14–16^. Despite these efforts, the forest is still being illegally deforested and replaced with pastures and soy^17^.

To verify these illegal deforestations, there are two databases freely available in Brazil. The Amazonia Deforestation Monitoring Project (PRODES), which is developed and executed by the National Institute for Space Research (INPE), and the Amazonia Geoinformation Program (SAD/ImazonGeo) developed by the Institute for Man and the Environment of the Amazon (Imazon). They identify the deforested areas in Amazonia region from remote sensing (RS) techniques^18,19^. The data provided by these programs assist research and alert to the implementation of conservation policies. SR is also helpful in identifying crops, and cross-referencing this information can tell us where the forest has been replaced by agriculture.

The *Perpendicular Crop Enhancement Index* (PCEI), recently used in mapping soybean areas^20,21^, has been positively effective in monitoring soybean farming and has become an essential tool in monitoring the expansion of the crop in the face of deforestation in the Amazonia^17,22–24^. Monitoring anthropic impacts using the SR technique has been effective in environmental studies^20,25^.

This study aimed to verify the conversion of forest into soybean areas in disagreement with the SoyM and using the deforestation data from PRODES and ImazonGeo in the Amazonia portion found in the northern region of the State of Mato Grosso, which is the largest grain producer in Brazil.

## Results

To authenticate the data obtained, the trend tests applied to the evaluated variables were performed and are shown in Table 1. All variables quantified with the ImazonGeo monitoring system presented a significant increase trend by the Mann–Kendall test, i.e., there is a trend for increased deforestation over the years evaluated as well as an increase in polygons. The PRODES system also showed an upward trend for all variables except for the number of total polygons. Pettitt test identified the year 2013 as the likely point of change in the time series in cases of an trend to increase. The exception was the total deforestation variable quantified by the PRODES system, where no point of change was identified.

**Table 1:** P-value of the Mann–Kendall and Pettitt tests for the variables total deforestation, deforestation for soybean planting, total polygons, and polygons for soybean planting obtained from the Imazon and PRODES monitoring systems.

Variable	Imazon			PRODES		
	Mann–Kendall	Pettitt	Year	Mann–Kendall	Pettitt	Year
Total deforestation	< 0.00	0.03	2013	0.03	0.19	–
Deforestation for soybean	< 0.00	0.03	2013	< 0.00	0.03	2013
Total polygons	< 0.00	0.03	2013	0.99	0.97	–
Polygons for soybean	< 0.00	0.03	2013	< 0.00	0.03	2013

**Figure 1:**
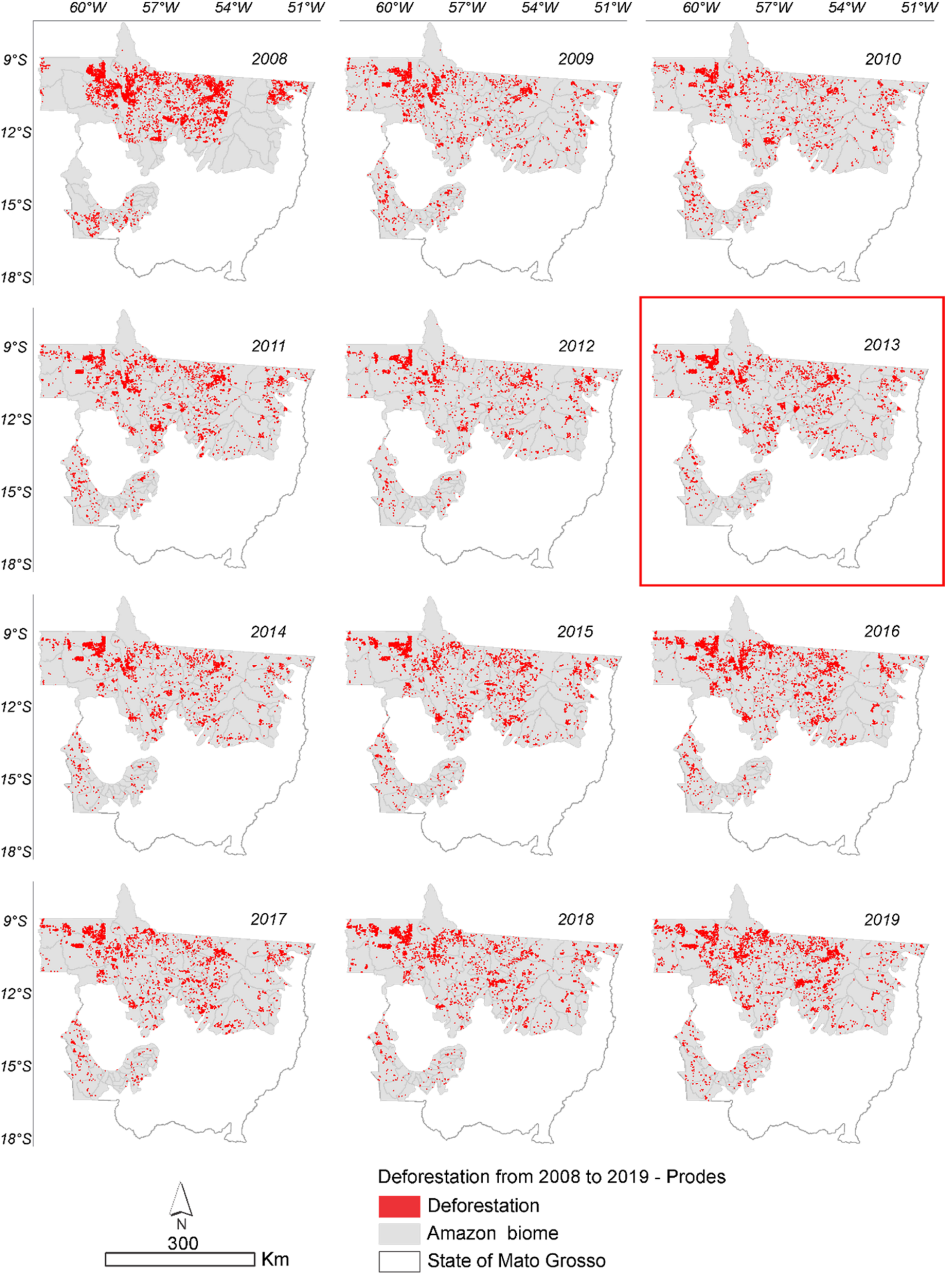
Annual deforestation in the Amazonia biome in the State of Mato Grosso from August 2008 to October 2019 with PRODES data. The highlighted year corresponds to the point of change in the time series through Pettitt test (Table 1).

**Figure 2:**
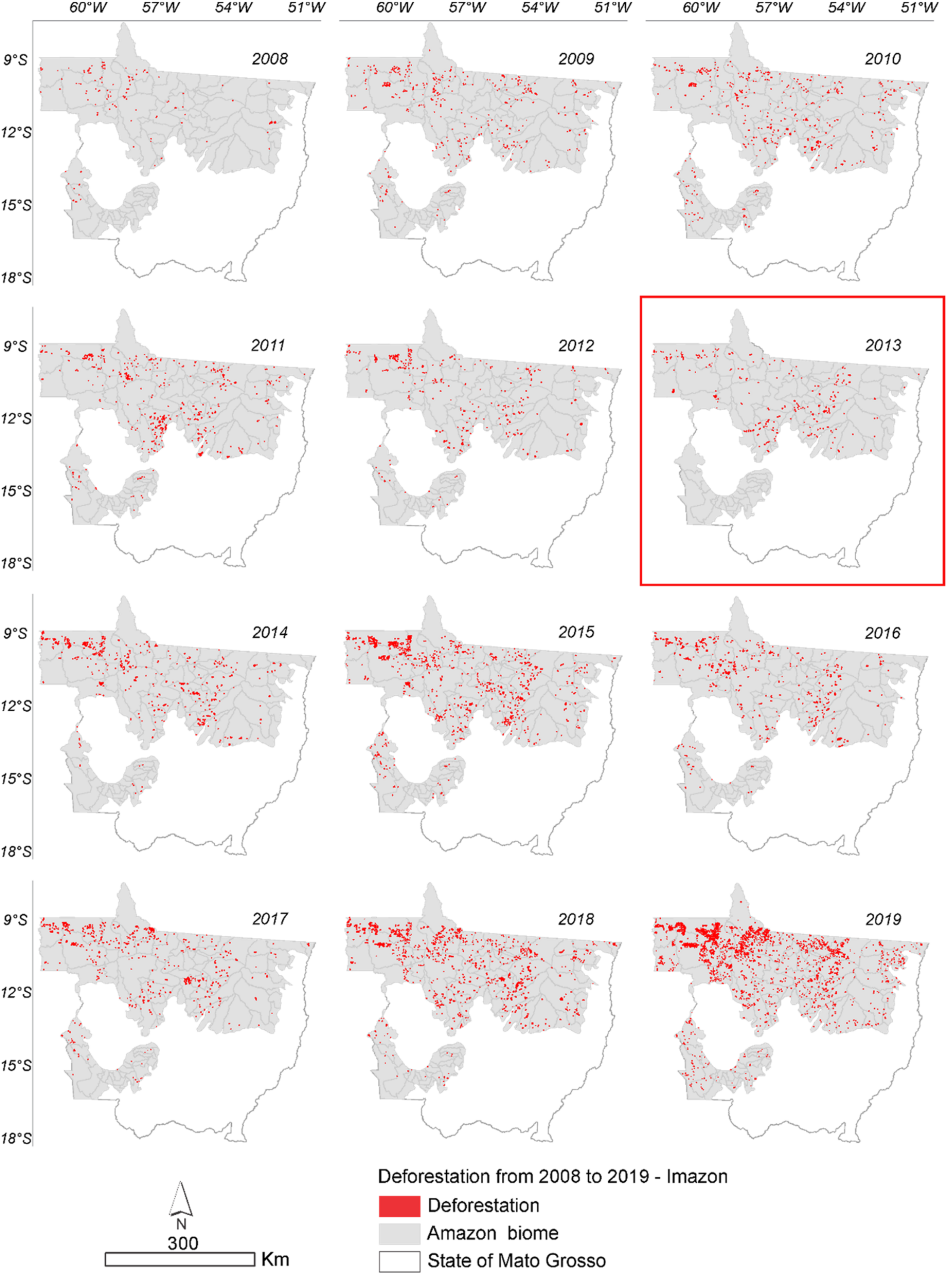
Annual deforestation in the Amazonia biome in the State of Mato Grosso from August 2008 to October 2019 with data from ImazonGeo. The highlighted year corresponds to the likely point of change in the time series identified by the Pettitt test (Table 1).

The results of the statistical analyses can be seen in Figs. 1 and 2, in which the year 2013 presented a change point, that is, an increase in deforestation. The results show that the deforested area in the Amazonia portion of the State of Mato Grosso between August 2008 to October 2019 has increased over the 12 years evaluated (Supplementary Table 1).

**Figure 3:**
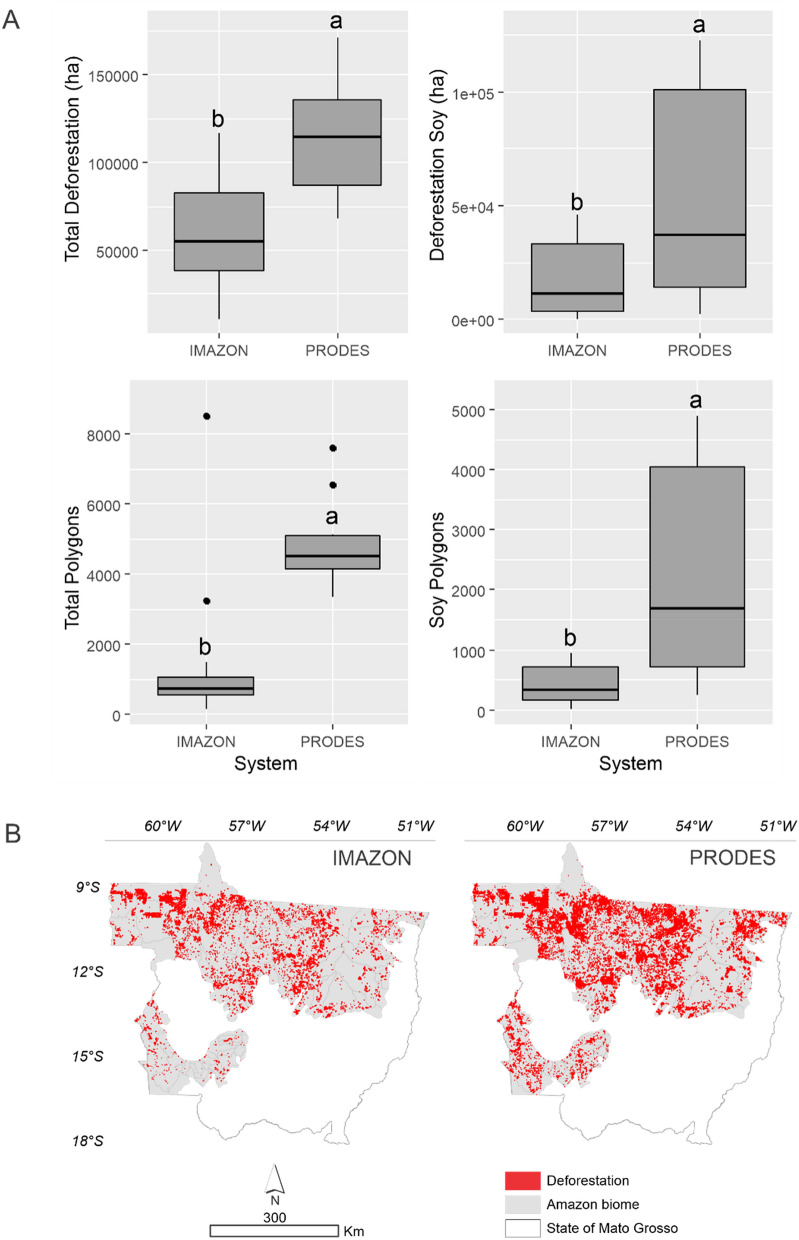
Boxplot for the variables total deforestation, deforestation for soybean planting, total polygons, and polygons for soybean planting obtained with the Imazon and PRODES monitoring systems (**A**). Means followed by different letters in the same figure differ from each other by the t-test at 5% probability and cumulative deforestation from August 2008–2019 by ImazonGeo and PRODES (**B**).

Figure 3(A) shows the variation between variables over the time series as a function of the monitoring systems. It is possible to observe that the PRODES system presented higher variability in the data and statistically higher means than ImazonGeo in all cases.

**Figure 4:**
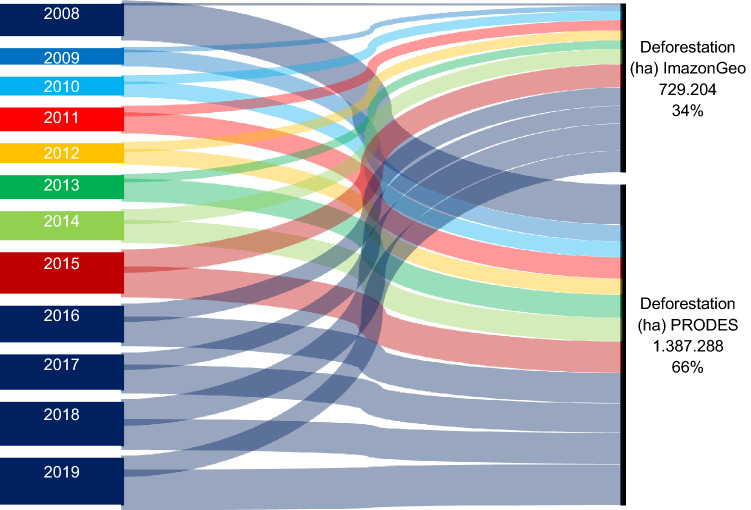
Sankey diagram showing, from the thickness of the lines and similar colors, the flows of deforestation variation over the years for PRODES and ImazonGeo during August 2008 to October 2019.

According to the data obtained from PRODES, the accumulated deforestation from August 2008 to the end of 2019 was 1,387,288 hectares, and for the ImazonGeo data, it was 729,204 hectares (Fig. 3B). Thus, when comparing the two databases from the Sankey diagram (Fig. 4), PRODES corresponded with the larger deforested area, with 66%, while ImazonGeo corresponded to 34%.

Soybean area detection analysis points to a total of 1,637,791 ha in 2008/2009, expanding to 4,303,791 ha in 2019/2020 (Supplementary Fig. 1). This finding represented an increase of 2,666,000 ha or a 2.6 times expansion of the planted area in this short period.

**Figure 5:**
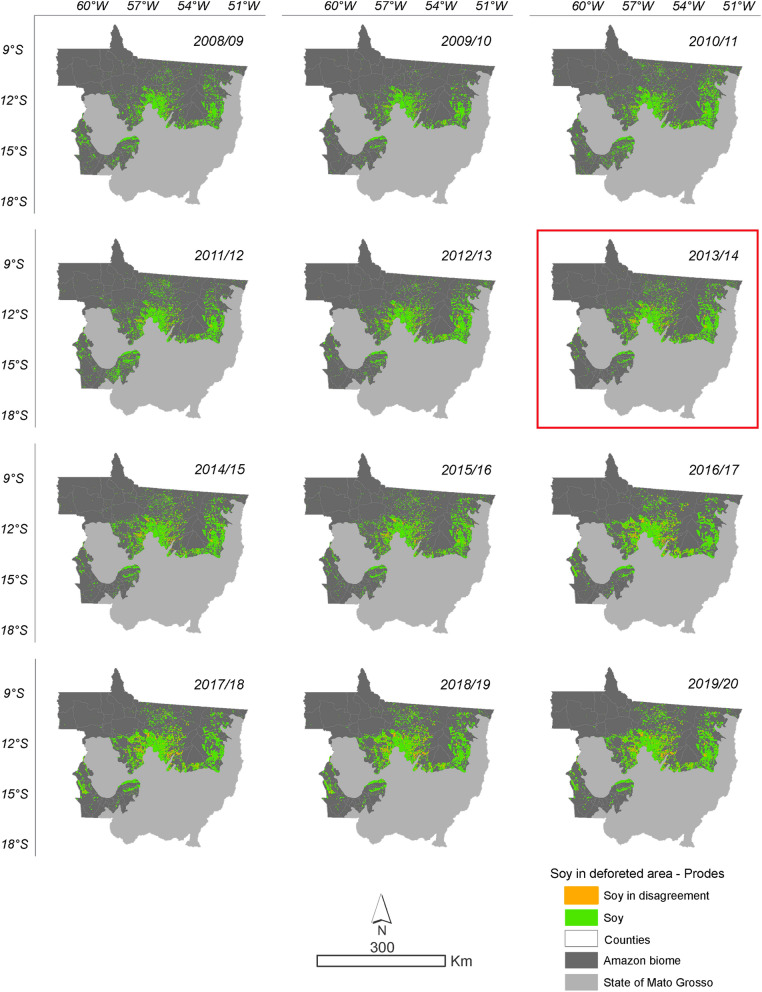
Soybean planted in the deforested area according to PRODES deforestation data. The highlighted year corresponds to the likely change point in the time series identified by the Pettitt test (Table 1).

**Figure 6:**
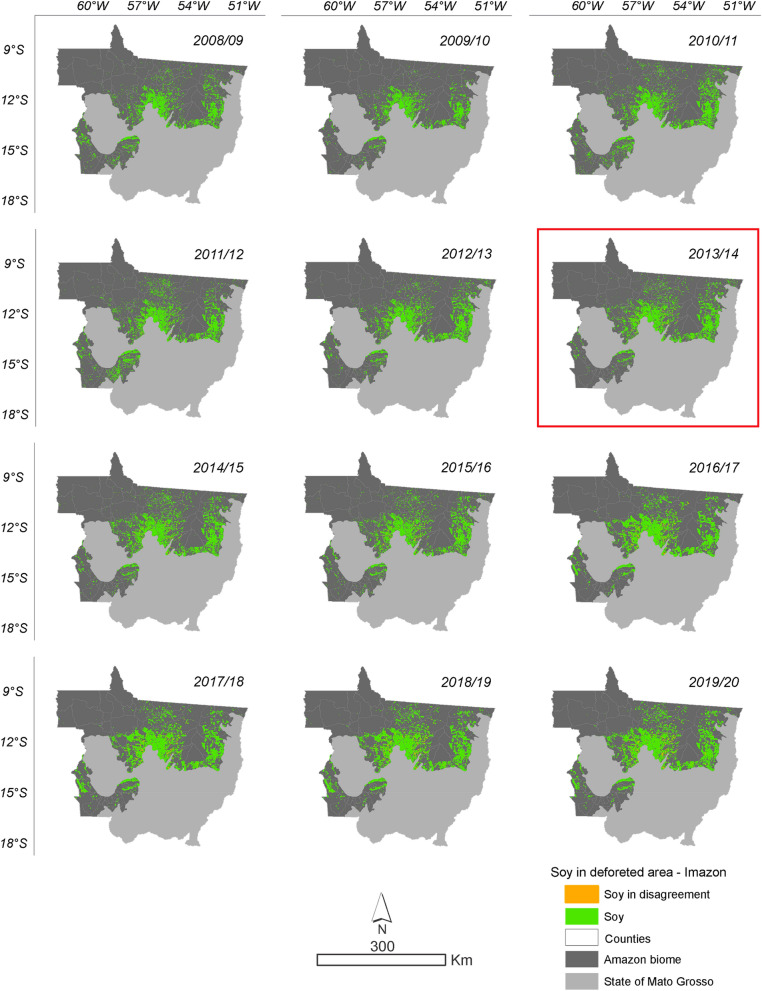
Soybean planted in the deforested area according to ImazonGeo deforestation data. The highlighted year corresponds to the likely point of change in the time series identified by the Pettitt test (Table 1).

**Figure 7:**
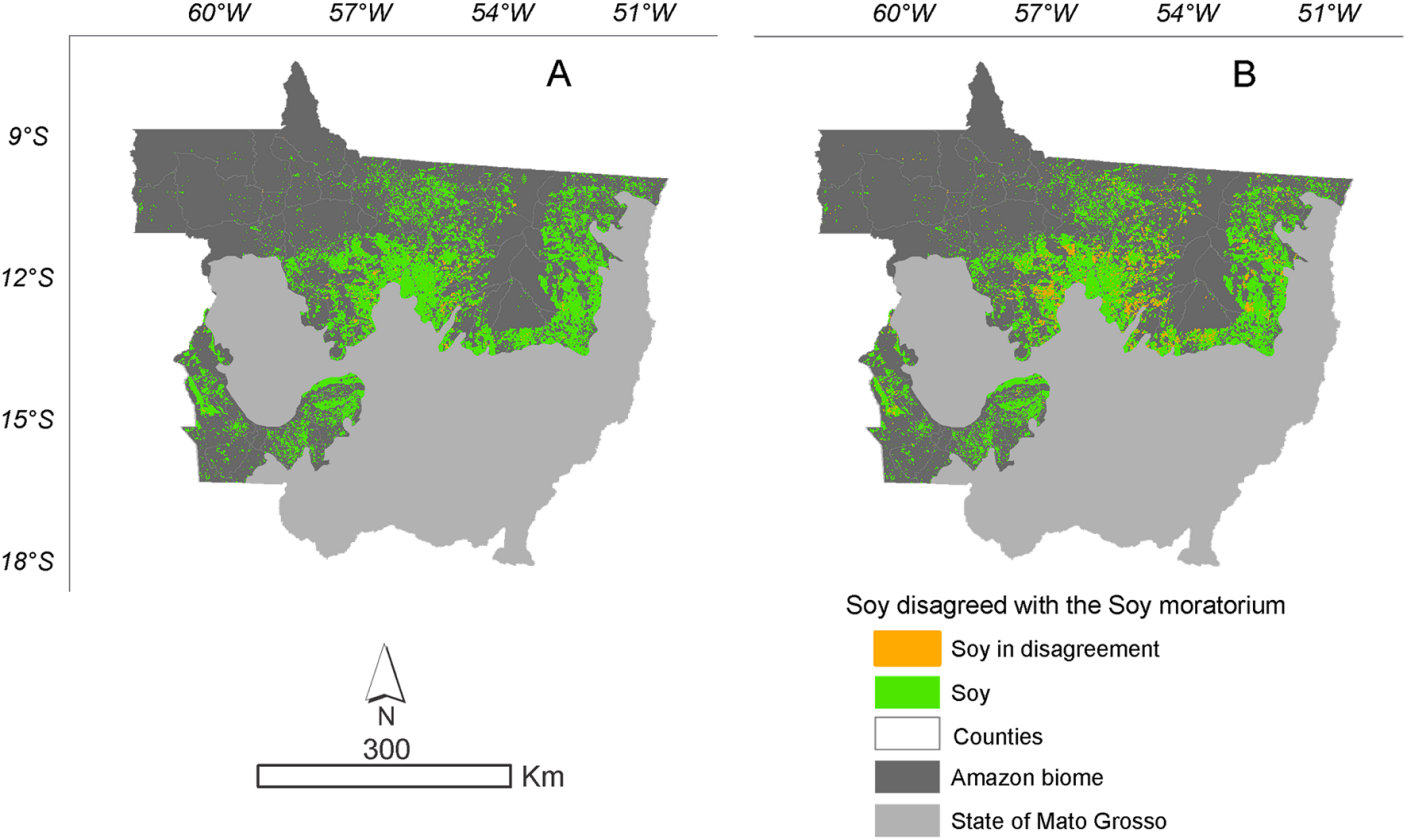
Accumulation of soybean planted in deforested area. (**A**) Soybean planted in deforested area according to ImazonGeo deforestation data. (**B**) Soybean planted in deforested area according to PRODES deforestation data.

According to the data obtained from the intersect between deforestation and soybean areas, it is evident that the conversion of forest to soybean areas has increased during the 2008/2009 to 2019/2020 crop seasons (Figs. 5 and 6). In the total time series evaluated by PRODES, 108,411 ha were converted into soybean areas by the 2019/2020 crop season, representing 7.81%. By ImazonGeo, a total of 46,253 ha were transformed into soybean areas by the 2019/2020 season, representing 6.3% (Supplementary Table 2). The soybean areas accumulated over the time series in disagreement with SoyM for PRODES and ImazonGeo are shown in Fig. 7.

The evolution of soybeans in disagreement with the SoyM in relation to the area planted with soybeans in the Mato Grosso Amazonia has increased during the crop years. In 2008/2009, it represented only 0.05% of deforestation converted into soybean areas, increasing to 2.51% of soybeans in disagreement with the SoyM in the 2019/2020 crop season, according to data obtained from the relationship between PRODES deforestation and soybean areas (Supplementary Table 3). The same happened with the relationship obtained with the deforestation data from ImazonGeo and areas of soybean cultivation. In the 2008/2009 crop season, there were 0.004% of soybean in disagreement with the SoyM and reaching 1.07% in the 2019/2020 crop season (Supplementary Table 3). The evolution represented by soybean areas in hectares makes it evident that the use of the MODIS/Terra-Aqua sensor underestimates the monitoring of cultivated areas, in which it can be seen from the 2016/2017 crop year monitoring with more refined spatial resolution and with the precise detection of areas via MSI (10 m) and OLI (30 m) sensors in Google Earth Engine. Currently, the soybean areas mapped and refined with the new sensors and that count as part of this study can be accessed on the website (https://pesquisa.unemat.br/gaaf/plataformas/).

Soybean in disagreement with SoyM over the years relative to deforested area to the entire time series went from 0.07% in 2008 to 7.81% in 2019 for PRODES deforestation, while for ImazonGeo evolved from 0.01% in 2008 to 6.34% in 2019 (Supplementary Table 2).

The municipalities of Feliz Natal, Tabaporã, Nova Ubiratã, and União do Sul present the largest areas in disagreement with the SoyM in the analysis with data from PRODES and ImazonGeo (Supplementary Tables 4 and 5).

The municipality of Feliz Natal according to PRODES obtained deforestation of 51,496 ha over the years evaluated. Of these, 11,169 ha were occupied with soybean in disagreement, representing 21.68% of soybean over the deforested area. Next is the municipality of Tabaporã with 24,038 ha, of which 9,865 ha were converted to soybean, equivalent to 41.03% of soybean over deforested area. The first two municipalities with the largest areas in disagreement with the SoyM according to ImazonGeo data were Feliz Natal, whose deforested area was 36,298 ha and occupied by soybean was 6,157 ha, representing 16.97% of soybean in disagreement with the SoyM. The second municipality was Nova Ubiratã with 15,697 ha, of which 4,786 were converted to soybean, representing 30.48% in disagreement with the SoyM.

Supplementary Tables 4 and 5 show the ranking of the first ten municipalities in the State of Mato Grosso in disagreement with SoyM for PRODES and ImazonGeo data. The first ten municipalities in the ranking in disagreement with the SoyM for the PRODES data represented 72.12% of the forest areas converted to soybeans crop. For the ImazonGeo data, the first ten municipalities of the ranking represented 80% of the soybean in disagreement with the SoyM.

**Figure 8:**
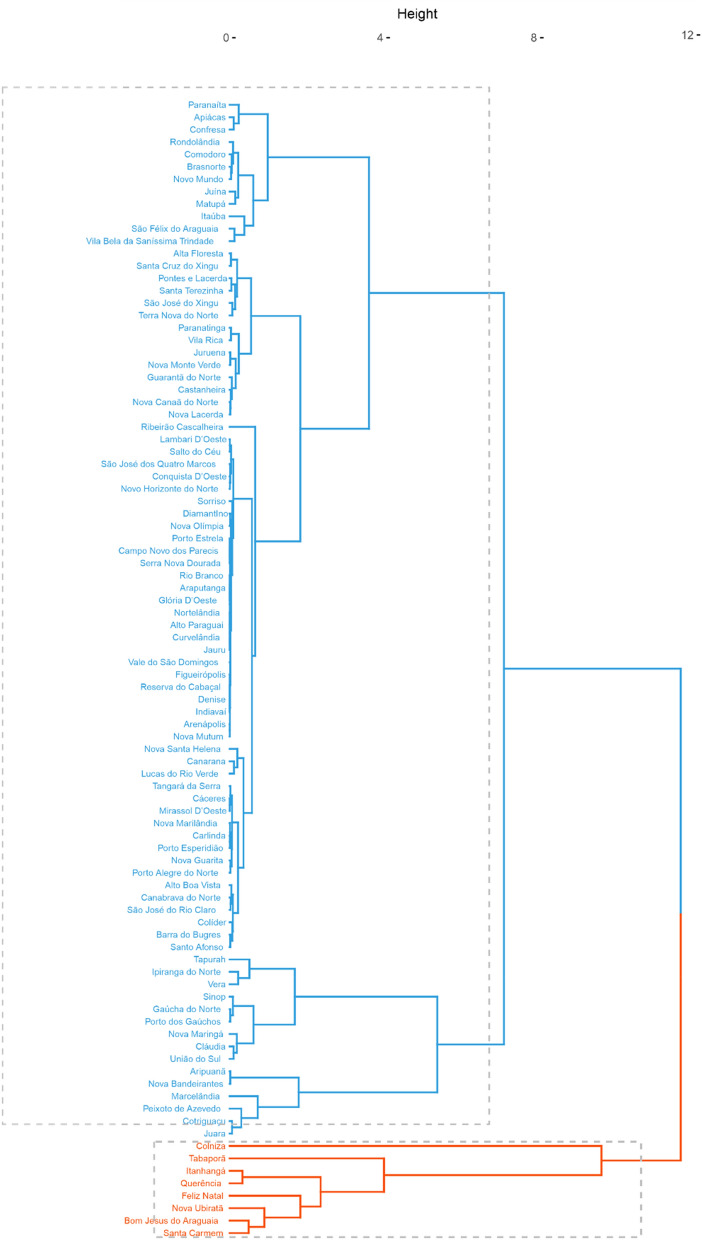
Cluster analysis of the municipalities of Mato Grosso based on the data of total deforestation and deforestation for soybean cultivation obtained by the PRODES system.

**Figure 9:**
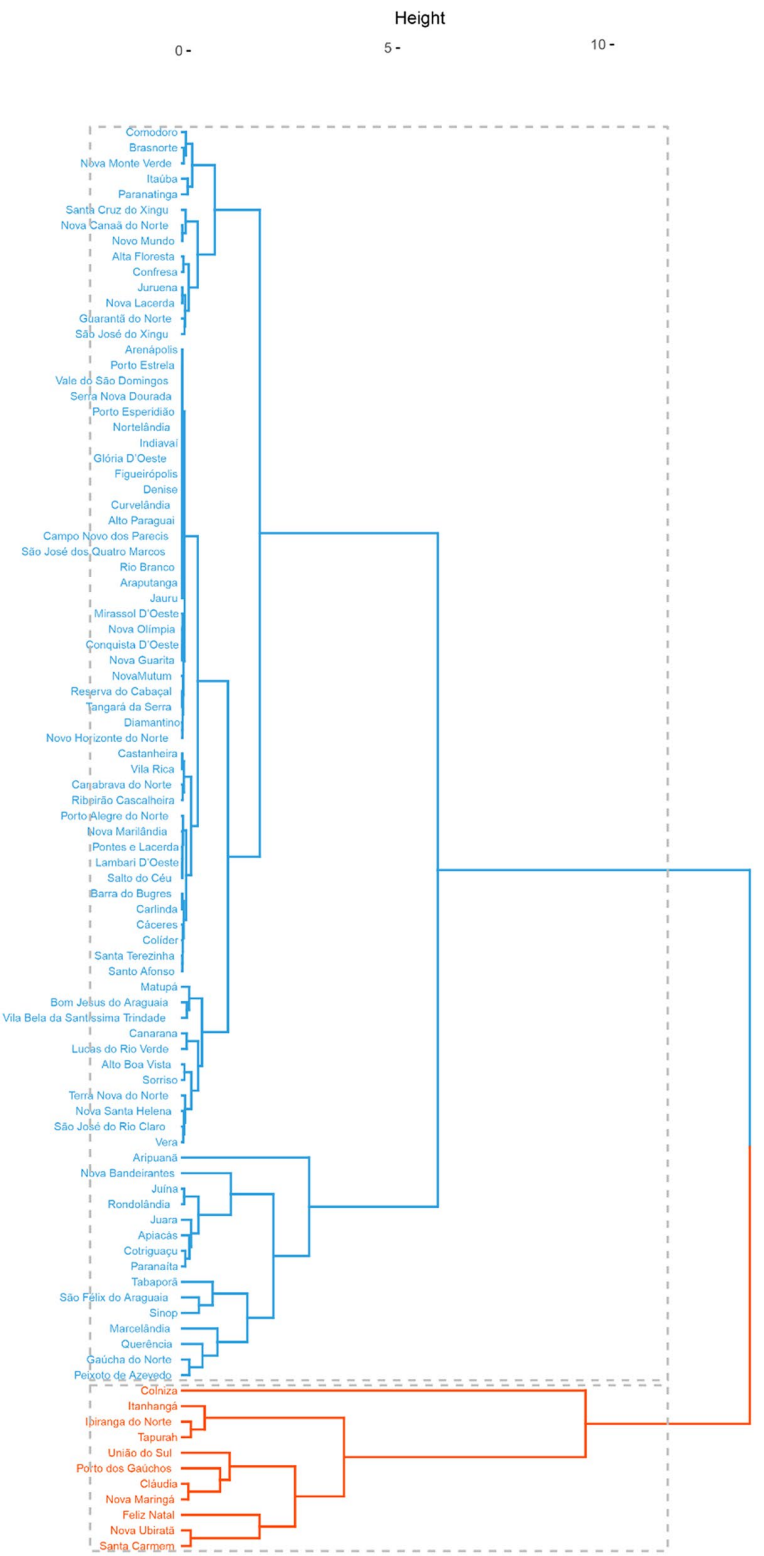
Cluster analysis of the municipalities of Mato Grosso based on the data of total deforestation and deforestation for soybean cultivation obtained by the ImazonGeo system.

Figures 8 and 9 show the cluster analysis of the municipalities of Mato Grosso based on the data of total deforestation and deforestation for soybean cultivation obtained by the ImazonGeo and PRODES monitoring systems, respectively. In both figures, the cluster in red contains the municipalities with the largest areas for the variables used. The municipalities in common in this group according to both systems were Colniza, Feliz Natal, Itanhangá, Nova Ubiratã, and Santa Carmem.

**Figure 10:**
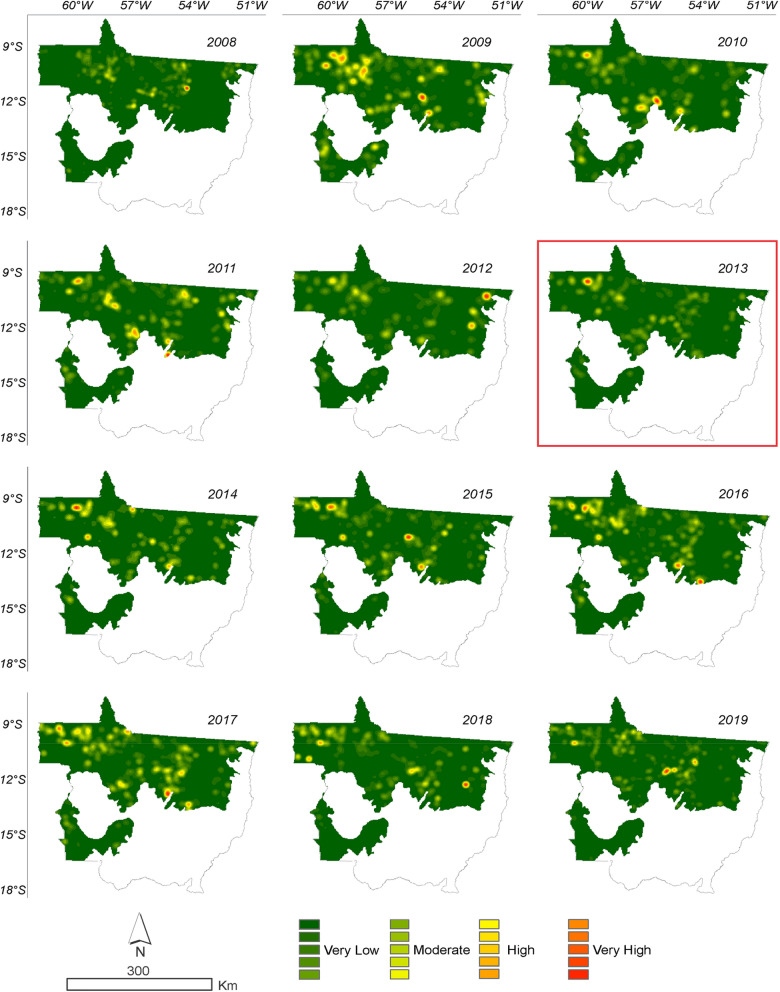
Annual deforestation density in the Amazonia biome in the State of Mato Grosso from 2008 to 2019 with data from PRODES. The year highlighted by the red square corresponds to the likely change point in the time series identified by the Pettitt test (Table 1).

**Figure 11:**
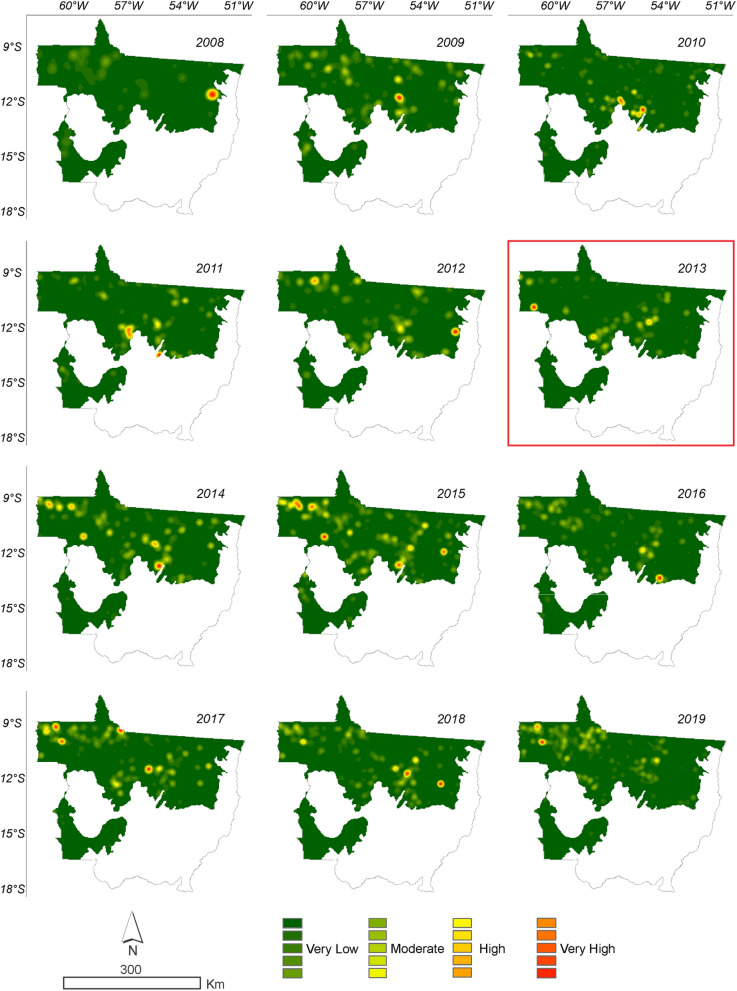
Annual deforestation density in the Amazonia biome in the State of Mato Grosso from 2008 to 2019 with data from ImazonGeo. The year highlighted by the red square corresponds to the likely change point in the time series identified by the Pettitt test (Table 1).

Based on the deforestation polygons presented in the maps of Figs. 2 and 3, it was possible to identify the regions of largest deforestation using the Kernel Density estimator as shown in Figs. 10 and 11.

**Figure 12:**
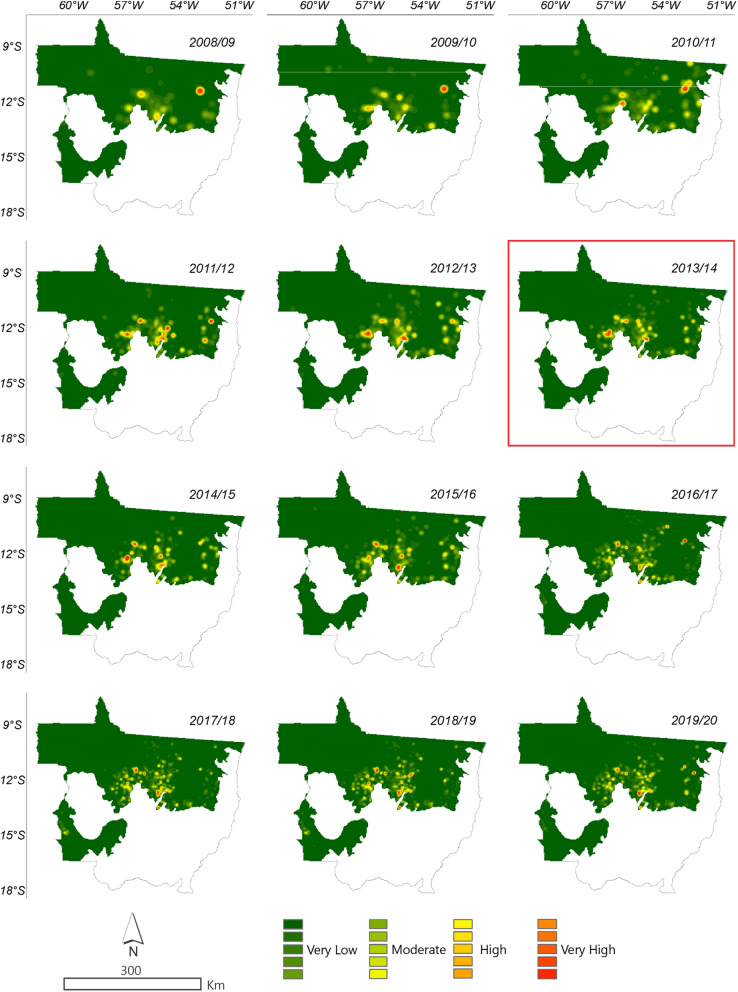
Density of deforestation caused by soybean in Amazonia biome in the State of Mato Grosso from 2008 to 2019 with data from PRODES. The year highlighted with red square corresponds to the likely point of change in the time series identified by the Pettitt test (Table 1).

It can be seen in Figs. 11 and 12 that the deforestation density over the time series is high and very high in all years evaluated for the PRODES and ImazonGeo data. More intense red and orange shades indicate the occurrence of a deforestation hotspot.

Figure 11 shows that in 2008 the region that presented the highest occurrence of deforestation was the municipality of Marcelândia. In 2009 were Santa Carmem to the south and Cláudia to the north. In 2017, we found a hotspot areas between the border of the municipalities of Cláudia, Itaúba, and Sinop, between the border of Apiacás and Paranaíta, and another area in Aripuanã and Colniza. In 2018, União do Sul and Querência presented the most intense hotspot areas, followed by 2019 with the municipalities of Colniza and Aripuanã.

**Figure 13:**
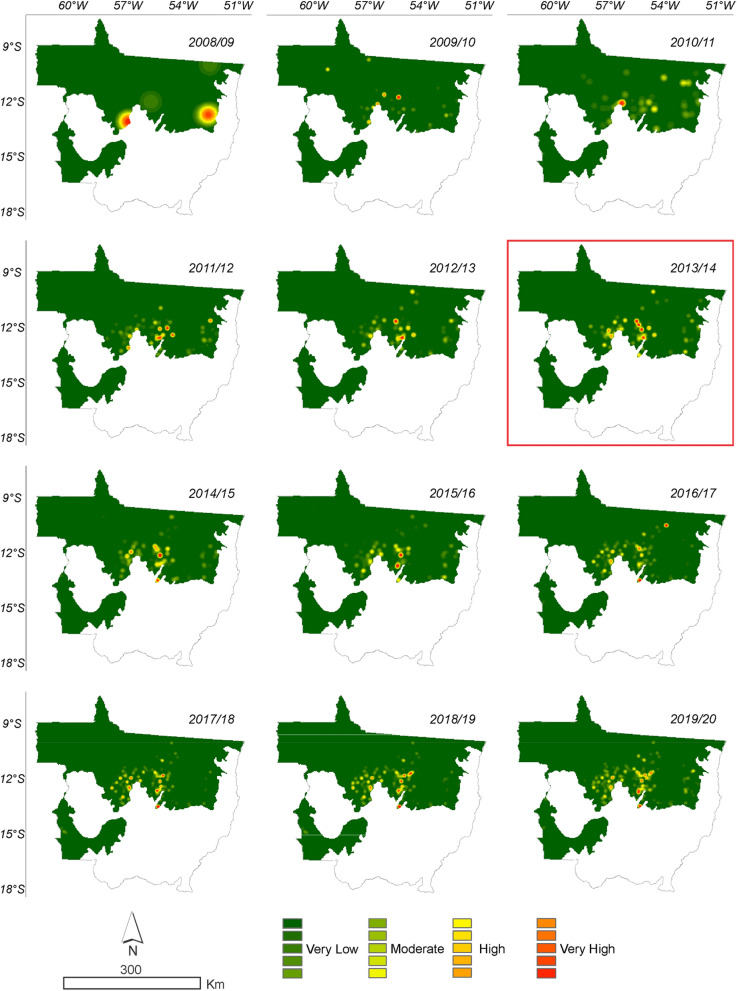
Density of deforestation caused by soybean in Amazonia biome in the State of Mato Grosso from 2008 to 2019 with data from ImazonGeo. The year highlighted with red square corresponds to the likely point of change in the time series identified by the Pettitt test (Table 1).

According to ImazonGeo data (Fig. 12) for deforestation in 2008, the region with the highest occurrence of deforestation was between the municipality of Canabrava do Norte and São Félix do Araguaia. In 2011 the municipalities of Nova Ubiratã and Itanhangá were the regions with the highest intensity. In 2012, the municipalities of Alto Boa Vista, Bom Jesus, and Colniza, in 2013 Rondolândia, and in 2014 the municipalities of Colniza, Juína and Cláudia were the regions with the highest deforestation. By evaluating deforestation for soybean using the KD estimator, it was also possible to identify the regions of higher occurrences in disagreement with the SoyM. Figures 12 and 13 show the regions where there were higher conversions of forest to soybean, that is, the more intense the red and orange tones indicate the occurrence of soybean in disagreement with the moratorium.

In Fig. 12, the municipalities that presented areas of intense occurrence, i.e., hotspots in disagreement with the SoyM according to the PRODES data in the 2008/2009 and 2009/2010 seasons was São Félix do Araguaia, followed in the 2010/2011 season in Ipiranga do Norte, Sorriso, and São Félix do Araguaia. In 2015/2016, the municipalities of intense occurrence that stood out were Tabaporâ, Feliz Natal, and Santa Carmem, followed by the municipality of São Félix do Araguaia in the 2016/2017 crop season. In the 2017/2018, 2018/2019, and 2019/2020 seasons, the occurrence of soybean areas in disagreement with the moratorium occurred with higher intensity in the municipalities of Tabaporã, Feliz Natal, Nova Ubiratã, União do Sul, and São Félix do Araguaia.

In Fig. 13 the municipalities that presented areas of hostspots in disagreement with the SoyM according to ImazonGeo data in the 2008/2009 season were Nova Mutum, Tapurah, Nova Maringá, São José do Rio Claro, Lucas do Rio Verde, Querência, Ribeirão Cascalheira, and Bom Jesus do Araguaia, followed in the 2009/2010 season by the municipality of Claúdia. In the 2015/2016 season, the municipalities of Santa Carmem and Feliz Natal stood out, followed by Nova Ubiratã and Peixoto de Azevedo in 2016/2017. In 2017/2018, 2018/2019, and 2019/2020, the municipalities of Nova Ubiratã and Feliz Natal were the most significant.

The data from PRODES and ImazonGeo indicate that the regions of highest deforestation occurrences throughout the time series for soybean crop are located in the southern and eastern regions of the biome.

## Discussion

The State of Mato Grosso is the major commodity producer in Brazil^26,27^ and has one of the most active deforestation frontiers in the Amazonia^19^ due to the global demand for food and biofuels^28–30^. According to our findings, deforestation rates in Mato Grosso Amazonia from 2008 to 2019 were 1,386,497 ha for PRODES and 729,204 ha for ImazonGeo. Of this total, 1.97% of PRODES deforestation data occurred in indigenous lands (ILs) and 0.39% in conservation units (CUs), while 1.86% occurred in ILs and 0.67% in CUs according to ImazonGeo. One solution to contain and avoid deforestation was creating the first zero-deforestation agreement in the tropics, the soy moratorium (SoyM), aiming to prevent the purchase of soybeans produced in deforested areas after 2008^17,31^. However, the soybean area has increased in the State of Mato Grosso over the years. In 2019/2020, soybean occupied 10,650,421 ha in the State of Mato Grosso^24^, distributed in three biomes: Cerrado with 6,341,916 ha, Amazonia with 4,303,791 ha, and Pantanal with only 4,714 ha.

The detection analysis of soybean areas in Amazonia in the State of Mato Grosso point to 1,637,791 ha in 2008/2009, expanding to 4,303,791 ha in 2019/2020. These findings represented an expansion of 2.6 times the planted area in this short period. Despite soybean farming being one of the driving activities of the economy, the expansion of its areas implies environmental problems like deforestation, which can occur directly or indirectly when it occupies pasture areas, pushing the pastures to new areas^12,32–34^.

According to PRODES data regarding the soybean area planted in the Mato Grosso Amazonia, between the 2008/2009 season, only 0.05% of the crop occupied newly deforested areas, increasing to 2.51% in the 2019/2020 season, and the largest areas in disagreement with the SoyM were found in the municipalities of Feliz Natal, Tabaporã, Nova Ubiratã, and União do Sul. By the ImazonGeo data, the areas in disagreement evolved from 0.004% to 1.07% and the largest municipalities were Feliz Natal, Nova Ubiratã, União do Sul, and Nova Maringá. Expectation of new areas at odds with SoyM due to the environmental policies of the Brazilian government is that they will continue to occur in large soy-producing municipalities due to the high land value and limited availability of new areas. In municipalities where cattle raising predominates and soy cultivation is still in the consolidation phase, deforestation should not occur because there is a large offer of open pasture areas, degraded or not. When the area of soybean in disagreement in relation to total deforestation is evaluated, it becomes evident that the conversion of forest to soybean areas has increased from 2008/2009 to 2019/2020. Throughout the time series evaluated by PRODES, 108,411 ha were converted into soybean areas by 2019/2020 harvest, equivalent to 7.81%, while by ImazonGeo a total of 46,253 ha were converted into soybean areas by 2019/2020 harvest, representing 6.3%. Soybean in disagreement with the SoyM in relation to the deforested area throughout the time series went from 0.07% in 2008 to 7.81% in 2019 for the PRODES deforestation data, while it evolved from 0.01% in 2008 to 6.34% in 2019 for ImazonGeo data.

Silva Junior & Lima^17^ reported that soybean occupied 2.54% of the area deforested between August 2015 and July 2016 by PRODES data. Between 2004 and 2005, soybean resulting from deforestation was 30% falling to 1% in 2014^15^. These reports suggest that the SoyM was efficient in the early years and continues to be so, despite the progressive increase in the rates of non-compliant areas verified in this study. The Pettitt test applied to the time series identified 2013 as the likely point of change in the evaluated time series. This finding corroborates studies carried out by INPE^19^, which reported an increase in deforestation from 0.5 million ha in 2012 to 0.7 million ha in 2017. Environmental policies applied since 2000, such as the expansion of protected areas, creation of real-time monitoring program (DETER) and the SoyM were not sufficient to contain deforestation from 2013, which can be justified due to land grabbing and deforestation in rural settlements^35^.

By evaluating time series for deforestation, Gollnow et al.^36^ found that the year 2013 was a change point with increasing deforestation trends. The authors also highlight that direct deforestation for soybean crops decreased after the implementation of SoyM. However, indirect deforestation within the property increased and has accounted for more than half of the deforestation associated with soybean expansion since 2013. Another evidence for increased deforestation is that the SoyM does not punish farmers for deforestation on their farms that are not converted into soybean, which encourages deforestation for other uses^15,36^.

Another related factor is the increase in the price appreciation of the soybean bag due to the global demand for food and biofuels^37^. In the 2019/2020 crop season, the soybean bag reached R$ 170.00 (US$ 31.40), similar to the fat cattle that reached R$ 171.50 in 2019 on the B3 (Official Brazilian Stock Exchange)^38^. These favorable market conditions and the lack of enforcement of illegal deforestation may justify the increasing rates of areas in disagreement with SoyM and deforestation by displacement on the property^15,39^.

The current government of Jair Bolsonaro is also a concern, as his actions reducing resources for enforcement agencies and other practices have enabled changes in land use and occupation in Amazonia^40^. Furthermore, the government has supported the farmers' movement, which criticizes SoyM, justifying that the Brazilian Forest Code is one of the most thorough on the planet, and for this reason, they do not need ONGs overseeing the sustainability of soybeans. The actions of President Bolsonaro's government favor the expansion of agriculture in Amazonia^40,41^. Possibly the farmers have expectations that current deforestation may be forgiven in the future, as occurred with the New Forest Code of 2012, which allowed the legalization of land illegally deforested up to the year 2008^42^.

Most papers assessing deforestation in Amazonia and SoyM made use of PRODES data. Gibbs et al.^15^, when evaluating SoyM in Brazil, used deforestation data provided by PRODES. By evaluating the soy moratorium in the 2016/2017 season in the State of Mato Grosso, Silva Junior & Lima^17^ used deforestation data also provided by PRODES. Lima et al.^23^, when identifying non-compliant soybean areas in Amazonian states, applied PRODES deforestation data. Rudorff et al.^43^ monitored deforested polygons mapped by PRODES to detect non-compliant soybean fields in municipalities of Amazonian states. West & Fearnside^44^, when evaluating Brazil's conservation reform and deforestation, used PRODES data to assess the development of the Action Plan for the Prevention and Control of Deforestation in the Legal Amazonia. From this perspective, the scientific community considers PRODES to be the greatest tropical forest monitoring program, and it has been an effective tool for this purpose^45^.

Few studies have been found referring to ImazonGeo, which is an important system in generating data that enables analysis by society, assisting in constructing and developing public policies^46–48^. The Mann–Kendall test results obtained here show a trend for the increase in deforestation over the years evaluated and an increase in polygons, except for polygons from PRODES. In all cases, the PRODES system presented greater data variability and statistically higher means than ImazonGeo. PRODES stands out in providing the largest deforested area, with 66% of deforestation, while ImazonGeo provided 34%. Even if the same sensor system collects the data from both programs, the methodology applied is different, so it will not have the same values. According to Maretto et al.^49^, the PRODES system depends on remote sensing experts to analyze the images from the sensor systems, which makes it an expensive and temporary task. Initiatives to automate image classification, as developed by Global Forest Watch and ImazonGeo, were carried out to minimize time and costs, but they were not as efficient as DETER and PRODES, which achieve accuracy of 90% in the classification^49,50^.

The PRODES monitoring program was identified with the most expressive result in the SoyM evaluation compared to ImazonGeo. A total deforested area of 1,386,497 ha was identified in the Mato Grosso Amazonia from August 2008 to 2019 for PRODES, while ImazonGeo identified 729,204 ha. PRODES stood out with the largest deforested area at 66% and ImazonGeo with 34%.

Even with the differences regarding the results presented in this study, it can be noted that there is a small percentage of soybean occupying deforested areas compared to the total area of soybean planted in the Mato Grosso Amazonia, which was 2.54% (PRODES) and 1.07% (ImazonGeo) with an increasing trend throughout the evaluated time series. Regarding the deforested area to the total of the time series went from 0.07% in 2008 to 7.81% in 2019 for the PRODES, and for ImazonGeo evolved from 0.01% in 2008 to 6.34% in 2019. Although these rates are low, what draws attention is that they have not been maintained over the years but have had a trend towards increasing, resulting from the public policies of the past and current governments.

## Material and methods

### Study area

**Figure 14:**
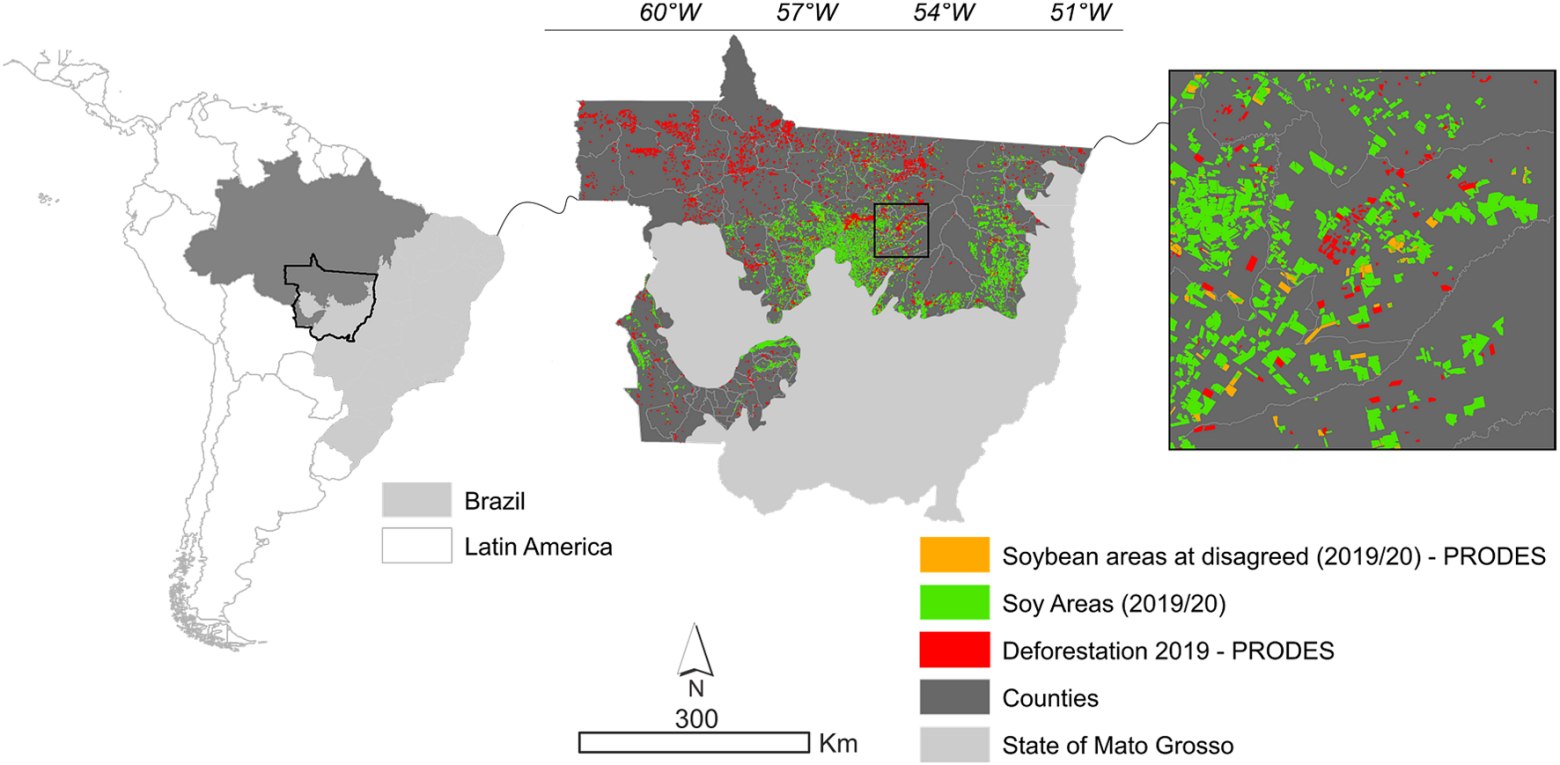
Study area comprising the Mato Grosso Amazonia biome.

The study area comprised the Amazonia biome in the State of Mato Grosso, located between 09º00’ to 18º00’S and 49º00’ to 61º00’W (Fig. 14). All the municipalities belonging to the Amazonia biome in the State were evaluated, covering an area of approximately 661 thousand km^2^ and encompassing 92 municipalities.

### Big data of spectral indices in soybean detection

The temporal intervals were selected based on the agricultural calendar of soybean cultivation in the State of Mato Grosso through the Google Earth Engine (GEE) platform^51^ for crop seasons from 2008/2009 to 2019/2020. A large amount of orbital data was used to perform the calculations of the PCEI index (Perpendicular Crop Enhancement Index—Eq. 1), developed by Silva Junior et al.^20,21^. The PCEI index uses reflectance factor values centered on the red and near-infrared wavelengths, ranging from -1 to 1, which allows for significant positive differences between the maximum and minimum observed in soybean crop phenology. For this purpose, we used the MODIS (Moderate Resolution Imaging Spectroradiometer), product MOD13Q1 V6 (MOD13Q1.006 Terra VegetationIndices 16-Day Global 250 m – Image Collection ID MODIS/006/MOD13Q1 and sur_refl_b01 (Red surface reflectance, 645 nm) and sur_refl_b02 (NIR surface reflectance, 858 nm)).

1 $$ {\text{PCEI}} = {\text{g}} \cdot \frac{{\left( {{\text{Max}}\frac{{\rho_{{{\text{IVP}}}} - a\rho_{{\text{V}}} - b}}{{\sqrt {1 + a^{2} } }} + {\text{S}}} \right) - \left( {{\text{Min}}\frac{{\rho_{{{\text{IVP}}}} - a\rho_{{\text{V}}} - b}}{{\sqrt {1 + a^{2} } }} + {\text{S}}} \right)}}{{\left( {{\text{Max}}\frac{{\rho_{{{\text{IVP}}}} - a\rho_{{\text{V}}} - b}}{{\sqrt {1 + a^{2} } }} + {\text{S}}} \right) + \left( {{\text{Min}}\frac{{\rho_{{{\text{IVP}}}} - a\rho_{{\text{V}}} - b}}{{\sqrt {1 + a^{2} } }} + {\text{S}}} \right)}} $$

wherein MaxPVI—maximum PVI value observed in the period of maximum soybean development; MinPVI—minimum PVI value observed in the pre-sowing and/or emergence period; S—enhancement coefficient (10^2^); g—gain factor (10^2^).

For the initial crop seasons, only the MODIS/Earth-Aqua sensor was used in an automated way in GEE, and from the crop season 2016/2017 on, we started the combination between MODIS, OLI (Landsat-8), and MSI (Sentinel-2) sensors. With the advancement of these sensors, we could further refine the soybean spatialization data due to the spatial resolution. The collections used in the combination of sensors were: USGS Landsat 8 Collection 1 Tier 1 and Real-Time data TOA Reflectance—LANDSAT/LC08/C01/T1_RT_TOA / B4 (Red, 0.64—0.67 µm) and B5 (Near infrared, 0.85—0.88 µm); and MultiSpectralInstrument, Level-1C—COPERNICUS/S2 / B4 (Red, 664.5 nm (S2A) / 665 nm (S2B)) and B8 (NIR, 835.1 nm (S2A) / 833 nm (S2B)). All medium-spatial resolution sensors used 20% of the pixel percentage clouds. In order to facilitate the process of separating the images and automating the crop monitoring, no specific date was used for each minimum and maximum PVI value. Instead, a period referring to a particular soybean stage was used, whereby only one image was generated with the best pixels from the images found in the stipulated time intervals by using *.filterDate()* and *median()* in the JavaScript language.

### Deforested areas data

Regarding deforestation data, PRODES (Legal Amazonia Deforestation Monitoring System) which provides annual deforestation estimates since 1988 based on Landsat satellite images at 30 m resolution with a minimum mapped area of 6.25 hectares^52,53^ and SAD/ImazonGeo data that uses MODIS images at 250 m resolution to detect areas larger than 10 hectares with subsequent validation on Landsat (30 m pixel) and CBERS (20 m pixel) satellite images^54^ were used to obtain the time series between August 2008 (Soy Moratorium agreement) until the year 2019. Then, the junctions of deforested areas in these years were carried out to verify the areas (polygons) converted from forest-soybean for all the municipalities belonging to the Amazonia biome in the State of Mato Grosso. All data were calculated using the South America Albers Equal Area Conic cartographic projection.

### Statistical analyses

Initially, boxplots were created to show the variation of the variables evaluated over the time series. A t-test was applied to compare the means of each monitoring system for each variable. The Mann–Kendall test was applied to verify the trend of the variables over the time series, followed by the Pettitt test to identify the likely point of change when the trend is significant. In all cases, a 5% probability level was adopted for the statistical tests performed.

#### Pettitt and Mann–Kendall tests

To identify trends over the time series (from August 2008–2019) of each variable, the Pettitt test was used. This non-parametric test allows confirmation of the stationarity of the historical series, i.e., observations are invariant concerning the chronology of their occurrences, except for random fluctuations.

Pettitt test locates the point at which an abrupt change in the mean of a time series occurred, and its significance was calculated by Eq. (2).

2 $$ p \cong 2\exp \left\{ {\frac{{ - 6k\left( T \right)^{2} }}{{\left( {T^{3} + T^{2} } \right)}}} \right\} $$

The point of abrupt change is *T*, where the maximum of *k*(*t*) occurs. The critical values of *k* are given by Eq. (3).

3 $$ k_{crit} = \pm \sqrt {\frac{{ - \ln \left( \frac{p}{2} \right)\left( {T^{3} + T^{2} } \right)}}{6}} $$

To analyze the time series trend of the aforementioned variables, the Mann–Kendall test will be applied (Mann, 1945; Kendall, 1975) (Eq. 4).

$$ {\text{Z}}_{{{\text{mk}}}} = \frac{{{\text{S}} - 1}}{{\sqrt {{\text{Var}}\left( {\text{S}} \right)} }};\quad {\text{when}}\;{\text{S}} > 0 $$

4 $$ {\text{Z}}_{{{\text{mk}}}} = 0;\quad {\text{when}}\;{\text{S}} = 0 $$

$$ {\text{Z}}_{{{\text{mk}}}} = \frac{{{\text{S}} + 1}}{{\sqrt {{\text{Var}}\left( {\text{S}} \right)} }};\quad {\text{when}}\;{\text{S}} < 0 $$

wherein Z_mk_ is the Z-index of the Mann–Kendall test; S is the "score" statistic, and Var (S) is the variance of the S statistic.

#### Sankey diagram

Sankey diagrams, which act as a visual accounting system between two or more variables having a starting point and at least one ending point illustrating the flows, were constructed. The thickness of each line is proportional to the flow magnitude^55–57^. In this way, it allows the deforestation flow to be verified year by year for the PRODES and ImazonGeo monitoring programs.

#### Cluster analysis

Ward's hierarchical agglomerative algorithm (1963) was used, with the average Euclidean distance as the measure of dissimilarity^58,59^.

Clusters were generated using the total deforestation data (area in ha) and deforestation for soybean cultivation (area in ha) from each monitoring system. The Euclidean distance and Ward's hierarchical method were used for this purpose. All analyses were performed on the R software using the packages “ggplot2”, “trend”, “ManKendall”, and “factoextra”.

#### Kernel density

All the annual series of deforestation data and soybean substituting native forest, among the public data, were submitted to the use of the Kernel Density (KD) estimator to identify the cities/regions with the highest occurrences of deforestation and in disagreement with the Soy Moratorium^60^, obtained by Eq. (5):

5 $$ \hat{f}_{h} \left( x \right) = \frac{1}{nh}\mathop \sum \limits_{i = 1}^{n} K\left( {\frac{{x - X_{i} }}{h}} \right) $$

wherein K is the Kernel function $\int {K\left( s \right)} \;ds = 1$; h is the search radius; x is the position of the center of each cell in the output raster; *X*_*i*_ is the position of point *i* coming from the centroid of each polygon; and n is the total number of fire foci.

## Supplementary Information

Supplementary Information.
