# Supplementary material for: Advance of soy commodity in the southern Amazonia with deforestation via PRODES and ImazonGeo: a moratorium-based approach
Source: Sci Rep. 2021 Nov 8;11:21792. doi: 10.1038/s41598-021-01350-y (PMC8576044; doi:10.1038/s41598-021-01350-y)
Supplement: Supplementary file 1 — Supplementary Information. [file 41598_2021_1350_MOESM1_ESM.docx]

**Supplementary Files**

Table 1. Mapping deforestation (ha) in the Amazonia biome in the State of Mato Grosso from August 2008 to October 2019 according to the Amazonia monitoring programs PRODES and ImazonGeo.

|  | **Years** | | | | | | | | | | | |
| --- | --- | --- | --- | --- | --- | --- | --- | --- | --- | --- | --- | --- |
|  | **2008** | **2009** | **2010** | **2011** | **2012** | **2013** | **2014** | **2015** | **2016** | **2017** | **2018** | **2019** |
| **PRODES** | 174,377 | 71,956 | 68,111 | 91,846 | 70,402 | 100,130 | 101,344 | 136,598 | 131,331 | 127,336 | 135,028 | 178,038 |
| **ImazonGeo** | 10,908 | 22,328 | 39,279 | 44,388 | 42,809 | 37,034 | 66,027 | 101,398 | 79,600 | 76,185 | 116,684 | 92,565 |

Table 2. Areas in non-compliance with the Soy Moratorium in Amazonia biome, State of Mato Grosso, from August 2008 to October 2019 in relation to total deforestation according to the Amazonia monitoring programs PRODES and ImazonGeo.

| **Soybean areas in non-compliance with the Soy Moratorium regarding deforestation** | | | | | | | | | | | | |
| --- | --- | --- | --- | --- | --- | --- | --- | --- | --- | --- | --- | --- |
|  | **Total Deforastation - PRODES (ha)** | | | | | | **Total Deforastation - ImazonGeo (ha)** | | | | | |
|  | 1,387,288 | | | | | | 729,204 | | | | | |
| **Crop Seasons** | **2008/09** | **2009/10** | **2010/11** | **2011/12** | **2012/13** | **2013/14** | **2014/15** | **2015/16** | **2016/17** | **2017/18** | **2018/19** | **2019/20** |
| **Soy (ha) in non-compliance PRODES %** | 915 | 3.443 | 8.737 | 9.685 | 16.784 | 29.333 | 35.789 | 39.420 | 82.198 | 86.223 | 95.980 | 108.411 |
|  | 0.07% | 0.25% | 0.63% | 0.70% | 1.21% | 2.11% | 2.58% | 2.84% | 5.93% | 6.22% | 6.92% | 7.81% |
| **Soy (ha) in non-compliance ImazonGeo %** | 70 | 957 | 3.490 | 3.641 | 6.378 | 9.066 | 13.979 | 16.921 | 33.019 | 34.155 | 38.880 | 46.253 |
|  | 0.01% | 0.13% | 0.48% | 0.50% | 0.87% | 1.24% | 1.92% | 2.32% | 4.53% | 4.68% | 5.33% | 6.34% |

Table 3. Total soybean area in the Amazonia biome in the State of Mato Grosso (MT) and areas in disagreement with the Soy Moratorium according to PRODES and ImazonGeo.

| **Area (ha) in non-compliance with the Soy Moratorium** | | | | | | | | | | | | |
| --- | --- | --- | --- | --- | --- | --- | --- | --- | --- | --- | --- | --- |
| **Crop Seasons** | | | | | | | | | | | | |
|  | **2008/09** | **2009/10** | **2010/11** | **2011/12** | **2012/13** | **2013/14** | **2014/15** | **2015/16** | **2016/17** | **2017/18** | **2018/19** | **2019/20** |
| **Soybean area in Amazon - MT** | 1,637,791 | 1,490,180 | 2,058,822 | 1,998,959 | 2,131,340 | 2,219,721 | 2,352,522 | 2,155,030 | 4,018,569 | 4,132,488 | 4,276,559 | 4,303,791 |
| **Soybean in non-compliance (PRODES)** | 915 | 3,443 | 8,737 | 9,685 | 16,784 | 24,530 | 35,789 | 39,420 | 82,198 | 86,223 | 95,980 | 108,411 |
|  | (0.05%) | (0.24%) | (0.43%) | (0.48%) | (0.78%) | (1.10%) | (1.52%) | (1.82%) | (2.56%) | (2.05%) | (2.24%) | (2.51%) |
| **Soybean in non-compliance (ImazonGeo)** | 70 | 957 | 3,490 | 3,641 | 6,378 | 9,066 | 13,979 | 16,921 | 33,019 | 34,155 | 38,880 | 46,253 |
|  | (0.004%) | (0.07%) | (0.17%) | (0.18%) | (0.29%) | (0.40%) | (0.59%) | (0.78%) | (0.83%) | (0.83%) | (0.91%) | (1.07%) |

Table 4: Ranking of the ten municipalities in the State of Mato Grosso in non-compliance with the Soy Moratorium for the PRODES data.

| **Ranking** | **Municipalities** | **Amazonia area (ha)** | **Deforestation (ha) from 2008 to 2019 (PRODES)** | **Polygons of deforestation (nº) from 2008 to 2019 (PRODES)** | **Soybean cultivated (ha) in deforested area from 2008-2019 (PRODES)** | **Soybean polygons (nº) in non-compliance with the Soy Moratorium** |
| --- | --- | --- | --- | --- | --- | --- |
| 1 | Feliz Natal | 1,145,761 | 51,496 | 1,034 | 11,169 | 337 |
| 2 | Tabaporã | 844,807 | 24,038 | 791 | 9,865 | 395 |
| 3 | Nova Ubiratã | 761,327 | 23,757 | 730 | 9,420 | 401 |
| 4 | União do Sul | 458,194 | 30,332 | 766 | 8,093 | 227 |
| 5 | Porto dos Gaúchos | 686,216 | 22,821 | 525 | 7,230 | 264 |
| 6 | Itanhangá | 289,810 | 28,634 | 816 | 7,256 | 299 |
| 7 | Santa Carmem | 385,539 | 17,376 | 240 | 6,904 | 144 |
| 8 | Cláudia | 385,002 | 32,622 | 767 | 6,129 | 177 |
| 9 | Nova Maringá | 1,054,777 | 28,788 | 497 | 6,096 | 131 |
| 10 | Querência | 1,778,632 | 25,712 | 781 | 5,393 | 295 |
| **Total** | | **-** | **285,576** | **6,947** | **77,555** | **2,670** |

Table 5. Ranking of the ten municipalities in the State of Mato Grosso in non-compliance with the Soy Moratorium for the ImazonGeo data.

| **Ranking** | **Municipalities** | **Amazonia area (ha)** | **Deforestation (ha) from 2008 to 2019 (ImazonGeo)** | **Polygons of deforestation (nº) from 2008 to 2019 (ImazonGeo)** | **Soybean cultivated (ha) in deforested area from 2008-2019 (ImazonGeo)** | **Soybean polygons (nº) in non-compliance with the Soy Moratorium** |
| --- | --- | --- | --- | --- | --- | --- |
| 1 | Feliz Natal | 1,145,761 | 36,298 | 374 | 6,157 | 109 |
| 2 | Nova Ubiratã | 761,327 | 15,697 | 165 | 4,786 | 76 |
| 3 | União do Sul | 458,194 | 28,857 | 431 | 4,568 | 67 |
| 4 | Nova Maringá | 1,054,777 | 26,171 | 312 | 4,038 | 60 |
| 5 | Santa Carmem | 385,539 | 13,979 | 158 | 3,982 | 68 |
| 6 | Cláudia | 385,002 | 25,764 | 324 | 3,630 | 48 |
| 7 | Porto dos Gaúchos | 686,216 | 16,344 | 194 | 3,427 | 72 |
| 8 | Tapurah | 281,304 | 6,424 | 92 | 2,140 | 48 |
| 9 | Itanhangá | 289,810 | 12,082 | 158 | 2,092 | 47 |
| 10 | Ipiranga do Norte | 195,145 | 5,324 | 44 | 1,701 | 27 |
| **Total** | | **-** | **186,940** | **2,252** | **36,520** | **622** |


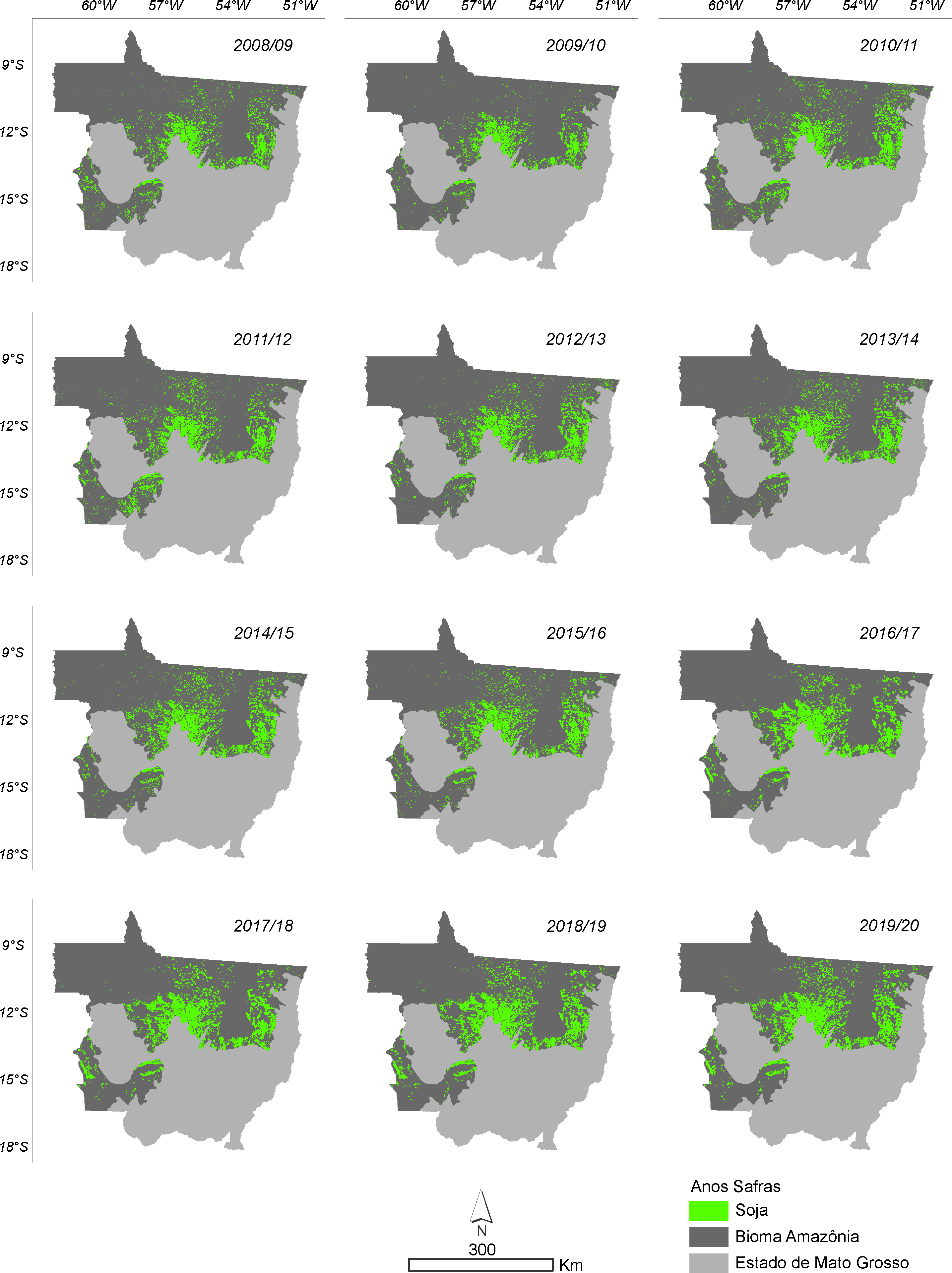


Figure 1. Soybean cultivated from 2008/2009 to 2019/2020 crop seasons in Amazonia biome in the State of Mato Grosso.
